# Composition of acacia honeys following processing, storage and adulteration

**DOI:** 10.1007/s13197-019-03587-y

**Published:** 2019-01-28

**Authors:** Nikolett Czipa, Clive J. C. Phillips, Béla Kovács

**Affiliations:** 1 Institute of Food Science, Faculty of Agricultural and Food Sciences and Environmental Management, University of Debrecen, Böszörményi Street 138, Debrecen, 4032 Hungary; 2 School of Veterinary Science, University of Queensland, Gatton, QLD 4343 Australia

**Keywords:** Acacia honey, Heating, Adulteration, Centrifugation, Collection

## Abstract

This study investigated the effect of different treatments (centrifugation and filtration; heating; adulteration with sugar syrups, and storage) and collection variables (year and region of the country) on the physicochemical properties of 44 Hungarian acacia honeys. The characteristics measured were diastase activity, hydroxyl-methyl-furfural (HMF), total phenolic content (TPC), electrical conductivity (EC), colour, pH, proline, moisture, sucrose, fructose and glucose contents, and concentration of eleven elements (As, B, Cd, Cr, Fe, K, Mg, Na, P, S, Zn). Centrifugation and filtration reduced the concentration of all examined parameters, except for moisture. Heating reduced diastase activity, proline and total phenolic concentrations and increased HMF concentration and colour value. Adulteration with sugar syrups had adverse effects on the diastase activity, proline, moisture and sugar concentrations, EC, colour and pH. Two-year storage reduced diastase activity, HMF, proline and TPC concentrations and increased sucrose concentrations. The collecting area influenced Na, Fe and As concentration, but the collecting year had no effect on the examined parameters. It is concluded that method and region of honey collection, duration of storage and processing all have major effects on the quality of acacia honey. Applied sugar syrup, although it affected honey quality, would be difficult to detect in the finished product.

## Introduction

Honey is a natural substance produced by *Apis mellifera* from flower nectar and honeydew. Hungary, which has favourable environmental and geographical conditions for honey production, made 24,000 tons in 2016 (KSH 2016), of which about 15,000 tons was exported. The most important honey types are from the flowers of acacia, sunflower, lime, silk grass and rape in Hungary. Honey is a very complex food and its properties depend on the botanical, environmental and treatment (storage, extraction techniques, etc.) conditions. In Hungary, the climatic and geographical conditions are excellent suitable for honey production. Honey is a good environmental indicator (Almeida-Silva et al. 2011) because the element content of soil, water and air in the collecting area can influence the element properties of honey. The determination of potentially toxic heavy metal concentrations is particularly important because of its relation to the environment. Electrical conductivity is also useful to determine because it has a strong correlation with mineral salts (Terrab et al. 2002), especially potassium (Guler et al. 2007). In the European Union, electrical conductivity in blossom honeys should have a maximum of 0.8 mS/cm (Council Directive 2001/110/EC, hereafter the EU Directive). Honey has an amino acid content of about 1% of dry matter, with the most important being proline (50–85% of total amino acids, Anklam 1998). A proline content of 180 mg/kg is the minimum value that is internationally recommended (Hermosín et al. 2003). According to Oddo and Bogdanov (2004), proline and electrical conductivity are the most important parameters to indicate the botanical origin of honey.

The three main enzymes in honey are diastase (amylase), invertase (sucrose) and glucose oxidase (Bogdanov et al. 2008). The quantity of diastase and invertase is variable in fresh honey and is indicative of the composition and concentration of nectar. Values also decrease after storage and heating (Tosi et al. 2008). According to the EU Directive the minimum diastase activity should be 8 DN.

Honey contains glucose, fructose, sucrose and about 25 different oligosaccharides (Doner 1977). In most honey types fructose prevails and glucose is the second most important sugar (Cavia et al. 2002). One of the most important sugars in relation to adulteration of honey is sucrose; however the content of this dissacharide is also influenced by the weather (Lampeitl and Franz 1997). According to EU Directive the minimum fructose and glucose content—determined as the sum of both in blossom honeys—is 60% and the maximum sucrose content is 10% in the case of false acacia.

Beekeepers often heat honey before packaging, with the reference temperature being 40 °C, even though higher temperatures may be applied. This reduces the specific gravity and increases ash content, pH, HMF, browning, phenolics and antioxidant activity. Hydroxy-methyl-furfural (HMF) is a cyclic aldehyde that is generated from fructose in the presence of acid. Fresh honey has zero or trace HMF, and the formation of HMF is very slow, as long as the temperature and period of storage is correct (White 2000). However, heating can accelerate the speed of formation, as can adulteration with invert sugar (Nozal et al. 2001). According to the EU Directive the maximum HMF content in honey is 40 mg/kg. Moisture content affects the storage life of honeys because too high a moisture content facilitates the fermentation process. According to the EU Directive the maximum moisture content is 20%.

The aims of this study were to: (1) determine the effect of centrifuging and filtration on the physicochemical parameters of acacia honeys; (2) determine the effect of heating on the physicochemical parameters of acacia honeys; (3) examine the effects of adulteration of acacia honeys samples with different sugar products; (4) examine the effects of collecting year and collecting area on the composition of acacia honey; (5) determine the effect of 2 years’ storage on the physicochemical parameters of acacia honeys.

## Materials and methods

### Samples

Forty-four acacia (*Robinia pseudoacacia*) honey samples, collected directly from beekeepers, were examined to determine physicochemical parameters: storage, centrifuging and filtration, collecting year and area, heating and adulteration with different sugar products. Three samples to be used for a study of the effects of centrifugation and filtration and one for studying adulteration and heating were collected in 2016. For examination of the effect of centrifuging and filtration, honey samples were collected from three counties of Hungary (Sample 1 from Hajdú-Bihar County, Sample 2 from Szabolcs-Szatmár-Bereg County and Sample 3 from Békés County). One–one honeycomb was chosen from each hive of these counties. After the removal of wax-capping the samples were taken directly from honeycomb, before individual centrifuging and filtration. For studying adulteration and heating one hive of Békés County was chosen and the sampling was carried out after the centrifuging and filtration of all of honeycombs from this hive. Inter-annual variations were examined using samples collected in 2014, 2015 and 2016. Five acacia honey samples collected in 2015 were used for the examination of 2-year storage. All samples (1000 g) were collected immediately after centrifuging and filtration in new, sterile glass jars and these samples were storage in the dark at room temperature (20 °C). Acacia pollens, wax cuppings and combs from beekeepers were also examined. Acacia pollens were collected directly from pollen traps, wax cupping and comb pieces were collected immediately from removal of wax cupping. Melissopalynological analysis was carried out to verify the botanical origin of honeys using the method described by MSZ 6950-3:1977 (microscopic analysis of honey) [13]. The acacia pollen proportion was higher than 50% in every examined honey sample.

### Treatments

The effects of centrifugation and filtration were examined with three acacia honey samples from three apiaries. From three hives in each, 1000 g honey samples were taken before and after centrifugation and filtration into new, sterile glass jars. Beside honey samples, acacia pollen grains, wax cuppings and combs were sampled from the same hives as the honey samples. Fresh acacia honey subsamples of 100 g were then placed into closed glass vessels followed by heating to 40, 50, 60, 70 or 80 °C for a 60 min period in a water bath (Bandelin Sonorex Digital DT 255H, Germany). Time measurement started when the temperature of the honey reached the required temperature.

Adulteration of the honey samples was with glucose syrup (GS), fructose-glucose syrup (FGS), invert sugar 1 (ISA) and invert sugar 2 (ISE). GS and FGS were obtained directly from the producer (Hungrana Ltd., Hungary). FGS was produced by hydrolysis of starch. GS is a purified and concentrated aqueous solution of polysaccharides, also produced by hydrolysis of starch. ISA was produced by acid hydrolysis of sucrose, with sugar, water and ascorbic acid heated together at 114 °C. ISE was produced by enzymatic hydrolysis with invertase enzyme (Sigma-Aldrich Kft., Budapest, Hungary) added to 70% sucrose solution. The enzyme was inactivated by heating at 80 °C for 10 min. The pure honey samples were then adulterated with GS, GFS, ISA and ISE at levels of 30% and 40%. The adulteration and the invert sugar (ISA and ISE) preparations were carried out in an accredited laboratory (ISO/IEC 17025:2005). Honey samples for examination of storage were kept in the dark at room temperature in closed glass vessels.

### Analytical methods

All chemicals were analytical grade or better. Ultrapure water was used to prepare of solutions and dilutions produced by a Milli-Q water purification system (Millipore S.A.S., Molsheim, France).

The digestion of samples for element analysis was carried out according to the method of Kovács et al. (1996), which has been validated using animal and plant materials in our accredited laboratory. A total of 3 g honey was added to 10 ml nitric acid (69% v/v; VWR International Ltd., Radnor, USA) and the samples allowed to stand overnight. Samples were pre-digested at 60 °C for 30 min. After cooling, 3 ml hydrogen-peroxide (30% v/v; VWR International Ltd., Radnor, USA) was added and the samples heated at 120 °C for 90 min. After digestion, ultrapure water was added to make a final volume of 50 ml. Samples were homogenized and filtered using qualitative filter papers (Sartorius Stedim Biotech S.A., Gottingen, Germany). The concentrations of boron, potassium, magnesium, sodium, phosphorus and sulphur were determined by ICP-OES (Inductively Coupled Plasma Optical Emission Spectrometer) (Thermo Scientific iCAP 6300, Cambridge, UK). The applied wavelengths were the following: 249.772 for B, 769.896 for K, 279.806 for Mg, 818.326 for Na, 213.617 for P and 182.563 for sulphur. Detection limits (DL) of ICP-OES were: 0.0004 mg/kg for B, 0.527 mg/kg for K, 0.104 mg/kg for Mg, 0.009 mg/kg for Na, 0.489 mg/kg for P and 0.108 mg/kg for S. The determination of arsenic, cadmium, chromium, iron and zinc was carried out using ICP-MS (Inductively Coupled Plasma Mass Spectrometry) (Thermo Scientific XSeries 2, Bremen, Germany). Measured isotopes (amu) were as follows: 75 for As, 111 for Cd, 52 for Cr, 56 for Fe and 66 for Zn. Detection limits (DL) of ICP-MS were: 0.019 µg/kg for As, 0.003 µg/kg for Cd, 0.38 µg/kg for Cr, 0.017 µg/kg for Fe and 0.004 µg/kg for Zn.

Moisture contents (in %) were determined by refractometry (AOAC 1995a, 969.38) using a Medline DIGIT 5890 ATC Honey Pocket refractometer (United Kingdom). Electrical conductivity (EC, in µS/cm) was determined according to the method of Bogdanov et al. (1997) in a 20% (w/v) solution of honey (in distilled water) using a conductometer (FiveEasy™ FE30, Mettler-Toledo AG, Switzerland). The pH values were measured in a 30% (w/v) solution of honey (in distilled water) with a pH meter (FiveEasy™ FE20, Mettler-Toledo AG, Switzerland), according to the MSZ 6943-3:1980 standard.

Diastase activity was determined following the spectroscopic method of Bogdanov (2009), using a spectrophotometer (Evolution 300 LC, Thermo Electron Corporation, England) at 660 nm. Applied reagents were iodine (VWR International BVBA, Leuven, Belgium), potassium iodide, sodium acetate, acetic-acid, sodium chloride and starch (Sigma-Aldrich Chemie GmbH, Steinheim, Germany). Diastase activity is expressed as a diastase number (DN). Determination of the hydroxyl-methyl-furfural (HMF) content of samples in mg/kg was based on the White method (Bogdanov 2009). Applied reagents were potassium hexacyanoferrate (II) trihydrate for Carrez I. and zinc acetate for Carrez II (VWR International BVBA, Leuven, Belgium) and sodium disulfite (AppliChem GmbH, Darmstadt, Germany). Proline content in mg/kg was measured using a spectrometric assay (Bogdanov 2009) with a spectrophotometer (Evolution 300 LC, Thermo Electron Corporation, England) at 510 nm. Applied reagents were formic acid (Sigma-Aldrich Chemie GmbH, Steinheim, Germany), ninhidrin, methoxyethanol, l-proline (Alfa Aesar GmbH & Co. KG, Karlsruhe, Germany) and 2-propanol (Sigma-Aldrich Chemie GmbH, Steinheim, Germany). TPC in mg gallic acid equivalent (GAE)/100 g was determined according to the Folin-Ciocalteu method (Meda et al. 2005). The absorbance of blue-colour complex was measured at 760 nm. Applied reagents were 3,4,5-trihydroxybenzoic acid (Alfa Aesar GmbH & Co. KG, Karlsruhe, Germany), sodium carbonate and methanol (Scharlab S.L., Spain), Folin-Ciocalteu reagent (VWR International S.A.S., France).

The colour of honey samples was determined by spectrophotometric measurement from a 50% (w/v) honey solution at 635 nm (White, 1984). The honeys were classified according to the Pfund scale after conversion of the absorbance values:

$$ {\text{mm}}\;{\text{Pfund}} = - 38.70 + 371.39 \times {\text{A}}_{635} $$

Sucrose, fructose and glucose contents were determined based on AOAC (1995b) 977.20 methods with HPLC. Applied chemicals were acetonitrile as a mobile phase (VWR International S.A.S., France), sucrose, fructose and glucose standard solution (Alfa Aesar, Thermo Fisher GmbH, Germany). Instruments and equipment used were a chromatograph (Merck Hitachi L6200A, Germany), detector (Merck LaChrom RI Detector L-7490, Germany), column (Phenomenex Luna 5µ NH2 100A, USA), sample clarification kit (PALL A/B Glass 13 m, Sigma-Aldrich Kft., Budapest, Hungary) and syringes (Hamilton MIcroliter^®^ # 710, Switzerland).

### Statistical analysis

Data were described using general terms (mean, standard deviation, minimum and maximum values), One-Way ANOVA (Linear Discriminant Analysis, LDA, and Least Significant Difference, LSD, tests), Independent-Sample T Tests and Pearson correlations, using SPSS for Windows Version 13 (SPSS Inc. Chicago, Illinois, USA). All analytical determinations were conducted in triplicate.

## Results and discussion

### Physicochemical parameters of acacia honey samples

Based on the statistical analysis (LSD test) the collecting years had no effect on the physicochemical parameters of honey samples. The measured values are summarized in Table 1. Juan-Borrás et al. (2014) measured very similar diastase activity (17.3 DN), electrical conductivity (190 µS/cm) and sugar content (2.20% for sucrose, 26.8% for glucose and 40.2% for fructose, respectively) in Spanish acacia honeys. Paszkowsky et al. (2014) also determined similar sugar content (< 0.5% for sucrose, 29.6% for glucose and 41.2% for fructose, respectively) and electrical conductivity (170 µS/cm) in Polish acacia honeys. Nayik et al. (2016) reported lower fructose and similar glucose and sucrose content (35.6 ± 1.8%, 31.7 ± 1.4% and 1.33 ± 0.09%, respectively) in Kashmir Valley acacia honeys. Turkish acacia honeys showed higher moisture (20.8 ± 2.555) and proline (282 ± 112 mg/kg) contents and electrical conductivity (300 ± 250 µS/cm), lower fructose and glucose contents (28.3 ± 3.2% and 24.2 ± 2.8%) and very similar TPC (16.0 ± 2.7 mg GAE/100 g) (Can et al. 2015). Anatolian acacia honeys showed lower TPC (9.80 ± 1.00 and 12.2 ± 1.10 mg GAE/100 g) and higher Fe concentrations (1.20 ± 0.10 and 1.40 ± 0.10 mg/kg) (Kaygusuz et al. 2016). Very similar B, Cr, Mg (3.7 ± 1.1 mg/kg, < DL, 5.0 ± 8.0 mg/kg) contents have been measured in Italian acacia honeys by Bontempo et al. (2015). Calabrian acacia honeys have produced much higher K (719 ± 390 mg/kg), Mg (70 ± 27 mg/kg), Na (91 ± 29 mg/kg), Fe (2.05 ± 0.72 mg/kg) and Cr (0.68 ± 0.25 mg/kg) concentrations (Di Bella et al. 2015).

**Table 1:** Minimum, maximum, mean and standard deviation values of measured parameters on the acacia honey samples (n = 44)

	Minimum	Maximum	Mean ± standard deviation
Diastase activity (DN)	15.2	20.5	18.4 ± 1.20
HMF content (mg/kg)	0.410	5.62	2.12 ± 1.43
Proline content (mg/kg)	204	293	245 ± 26
TPC (mgGAE/100 g)	10.5	22.1	16.5 ± 3.0
EC (µS/cm)	100	209	141 ± 34
Colour	Water white (< 9 mm)	White (25 mm)	Extra white (12 ± 5 mm)
pH	3.36	4.02	3.76 ± 0.21
Moisture (%)	17.0	19.8	18.4 ± 0.7
Sucrose (%)	< DL	2.21	0.45 ± 0.12
Fructose (%)	39.0	47.0	42.6 ± 2.53
Glucose (%)	27.3	33.0	29.7 ± 1.65
As (µg/kg)	6.65	36.5	13.8 ± 6.9
B (mg kg^−1^)	2.04	4.14	3.05 ± 0.51
Cd (µg/kg)	< DL	0.571	0.190 ± 0.142
Cr (µg/kg)	< DL	< DL	–
K (mg/kg)	132	237	185 ± 30
Fe (µg/kg)	25	584	279 ± 180
Mg (mg/kg)	2.48	10.2	5.09 ± 2.48
Na (mg/kg)	1.30	6.40	3.44 ± 1.16
P (mg/kg)	21.7	76.7	37.0 ± 11.8
S (mg/kg)	6.40	22.7	14.5 ± 3.4
Zn (mg/kg)	0.074	3.37	1.14 ± 0.96

In Hungary there exists a Regulation of Codex Alimentarius Hungaricus 2-100 Directive (2009), for “Honey with distinctive quality indication”. The specific requirements in this Regulation are: moisture content no more than 18.5%, sucrose content no more than 6%, HMF content no more than 20 mg/kg, ratio of glucose-fructose is 1.5–1.8, proline content is a minimum of 200 mg/kg, diastase activity is a minimum of 10 DN and the acacia pollen rate is min. 15%. From the examined 44 samples, 21 samples corresponded to these requirements.

Based on Pearson correlation coefficient (CC value), there were significant positive relationships at the 0.01 level between EC and K content (CC = 0.948), EC and Mg content (CC = 0.637), pH and As content (CC = − 0.557), K and Mg content (CC = 0.576), S and Mg content (CC = 0.672), fructose and glucose content (CC = 0.971). Lower CC values of between 0.4 and 0.5 (but still significant at the 0.01 level) were observed in case of EC and P (0.439), EC and Zn (0.475), pH and TPC (− 0.449), proline content and TPC (0.421), proline and K content (0.405), proline and P content (0.470), proline and S content (0.417), proline and Mg content (0.453), TPC and P content (0.434), K and P content (0.477), K and Fe content (0.411), K and Zn content (0.427), K and S content (0.408), Na and S content (0.428), S and Zn content (0.441), Fe and B content (0.428).

### The effect of centrifuging and filtration

After centrifuging and filtration the moisture content of samples increased (Table 2), which was probably due to hygroscopic absorption of moisture by the honeys following removal of the wax capping from the cells. The sugar content, diastase activity, HMF content and pH value did not change. Proline contents were reduced by 24% in samples 1 and 2 and 17.5% in sample 3. TPC was reduced in all three samples, by on average 40%. The reduction in proline and TPC contents can be explained by the filtration reducing the pollen content of samples, which had very high proline (11,711 ± 152 mg/kg) and TPC (484 ± 6 mg gallic acid equivalent [GAE]/100 g) contents. The reduction in electrical conductivity was about 80 µS/cm, probably due to the correlation of this parameter with the mineral content that decreased after the centrifuging and filtration. Before treatment samples were a white colour, but after centrifuging and filtration they were extra white. The reduction in macroelement concentration was significant, most importantly in potassium, sodium and phosphorus.

**Table 2:** Chemical parameters of acacia honey before and after centrifuging and filtration

	Sample 1		Sample 2		Sample 3	
	Before	After	Before	After	Before	After
Proline content (mg/kg)	374 ± 3.6	284 ± 3.0	287 ± 4	218 ± 3	229 ± 1	189 ± 2
TPC (mgGAE/100 g)	32.3 ± 0.1	20.5 ± 0.1	24.9 ± 0.3	14.8 ± 0.2	25.1 ± 0.1	14.9 ± 0.1
EC (µS/cm)	312 ± 1	224 ± 4	231 ± 3	157 ± 3	234 ± 4	153 ± 2
Colour (mm)	33.0 ± 1.0	12.0 ± 1	28.0 ± 1.5	17.0 ± 1.5	27.0 ± 1.0	12.0 ± 1
Moisture (%)	16.0 ± 0.01	16.5 ± 0.02	16.7 ± 0.1	18.4 ± 0.1	16.0 ± 0.1	17.5 ± 0.1
As (µg/kg)	22.7 ± 0.1	20.9 ± 1.0	11.0 ± 0.2	10.8 ± 0.5	13.6 ± 0.2	13.4 ± 0.5
B (mg/kg)	11.3 ± 0.1	5.39 ± 0.0	3.59 ± 0.02	2.94 ± 0.0	3.63 ± 0.16	2.85 ± 0.19
Cd (µg/kg)	< LOD	< LOD	< LOD	< LOD	< LOD	< LOD
Cr (µg/kg)	4.79 ± 0.02	< LOD	< LOD	< LOD	< LOD	< LOD
Fe (µg/kg)	774 ± 2	590 ± 2	598 ± 8	503 ± 9	749 ± 15	627 ± 7
K (mg/kg)	523 ± 1	367 ± 2	359 ± 1.7	221 ± 1.7	372 ± 1	208 ± 1
Mg (mg/kg)	8.14 ± 0.45	6.56 ± 0.15	4.42 ± 0.20	1.81 ± 0.13	5.61 ± 0.09	1.37 ± 0.22
Na (mg/kg)	13.8 ± 1.5	4.74 ± 0.11	12.3 ± 0.1	3.43 ± 0.02	9.85 ± 0.04	2.36 ± 0.05
P (mg/kg)	55.9 ± 1.2	37.6 ± 1.3	61.2 ± 0.85	46.3 ± 0.2	72.5 ± 0.4	44.7 ± 1.2
S (mg/kg)	34.2 ± 0.8	26.8 ± 1.4	32.8 ± 0.6	23.3 ± 0.6	31.6 ± 1.3	22.7 ± 1.4
Zn (µg/kg)	1014 ± 11	783 ± 9	723 ± 11	448 ± 5	891 ± 3	575 ± 7

*LOD* limit of detection

The main reason for these changes appears to be the presence of different extraneous material in the honey (e.g. wax capping, comb and bee pieces, pollens). After centrifuging and filtration these matters were removed by gravity and the filtering. In the case of Sample 1 (Table 2) the micro and macro element contents of comb and wax capping were determined. Both contained high concentrations of K, so this was much reduced after centrifuging and filtration (626 ± 15.1 mg K/kg in comb and 1018 ± 32.1 mg K/kg in wax capping). The reduction in phosphorus concentration was about 18 mg/kg, with 104 ± 1.54 mg/kg in the comb and 118 ± 2.16 mg/kg in the wax capping. The changes in boron and sodium concentrations were also important, but the content of these elements were not much increased in the comb (14.3 ± 0.34 mg/kg for boron and 19.0 ± 0.46 mg/kg for sodium) or the wax capping (14.7 ± 0.29 mg/kg for boron and 13.8 ± 0.32 mg/kg for sodium). Sulphur contents of comb and wax capping was similar in both (73.3 ± 1.32 and 71.6 ± 1.32 mg/kg in comb and wax capping, respectively). The change in magnesium concentration was negligible, although the content in comb (27.7 ± 3.36 mg/kg) and wax capping (32.3 ± 1.12 mg/kg) was high.

Sample 1 did not contain cadmium in either the comb or the wax capping. Before the centrifuging Sample 1 contained chromium, however afterwards the concentration of these elements was under the DL. The comb and wax capping contained chromium (35.6 ± 4.05 µg/kg and 62.1 ± 4.14 µg/kg), thus these elements were measurable in this sample before the centrifuging. After treatment these elements were removed from the honey. The arsenic content of Sample 1 came from the nectar, because this toxic element was not measurable in the comb or wax capping and its concentration did not change after the centrifuging. Very high iron and zinc concentrations were measured in the comb (10,110 ± 216 µg/kg and 12,028 ± 370 µg/kg) and wax capping (12,282 ± 49.9 µg/kg and 10,924 ± 118 µg/kg) that influenced the iron and zinc content of Sample 1. Another reason for the changed concentrations was the increase in moisture content of samples.

### The effect of heating

The results of thermal heating on the physicochemical properties of honey are presented in Table 3. Diastase activity was reduced by only 5 DN at 80 °C, which was probably because of the freshness of sample and because the initial activity was very high. The value of this parameter conformed to the EU Council Directive 2001/110/EC. Tosi et al. (2008) found that heating caused a much greater reduction in diastase activity (from 25.8 ± 0.9 to 14.1 ± DN) in six honey samples. Heating to 40 °C and 50 °C did not affect the HMF content, but the higher temperatures increased it. The values still did not exceed the limits of the EU Directive due to the initial low values. Singh and Bath (1997) reported that heating to 65 °C had a similar effect on the HMF content of their examined honey samples. In another study, heating to 70 °C for 60 min resulted in an increase in HMF content of sunflower and eucalyptus honeys; however these changes were not so pronounced as in our samples (Bath and Singh 1999). Singh and Bath (1998) reported that the honey with low pH value produced more HMF under heating. Our result confirmed this claim because our sample had a low pH value and the increase of HMF content was significant; even though the pH value did not change.

**Table 3:** The effect of heating to 40–80 °C for 60 min on the chemical parameters of acacia honeys

	Unheated	40 °C	50 °C	60 °C	70 °C	80 °C
Diastase activity (DN)	25.8 ± 0.3	25.4 ± 0.0	24.9 ± 0.1	24.6 ± 0.2	22.5 ± 0.1	20.7 ± 0.1
HMF content (mg/kg)	0.31 ± 0.01	0.29 ± 0.02	0.36 ± 0.05	4.45 ± 0.12	7.37 ± 0.26	10.2 ± 1.1
Proline content (mg/kg)	284 ± 3	280 ± 1	275 ± 1	272 ± 1	264 ± 1	253 ± 1
TPC (mgGAE/100 g)	20.5 ± 0.2	20.1 ± 0.8	18.8 ± 0.0	18.9 ± 0.2	17.8 ± 0.1	17.6 ± 0.6
EC (µS/cm)	224 ± 3	224 ± 4	219 ± 4	218 ± 2	219 ± 1	218 ± 3
pH	3.91 ± 0.01	3.99 ± 0.03	3.98 ± 0.03	4.02 ± 0.02	4.06 ± 0.03	3.96 ± 0.02
Moisture (%)	16.5 ± 0.1	16.5 ± 0.10	16.6 ± 0.1	16.6 ± 0.1	16.7 ± 0.2	16.6 ± 0.1
Colour (mm)	12.0 ± 0.2	15.7 ± 0.1	18.2 ± 0.3	20.3 ± 0.1	22.1 ± 0.2	23.8 ± 0.1

Heating reduced proline content, particularly at 70 °C and 80 °C, in which it was reduced by 18 and 31 mg/kg, respectively. Based on our previous study (Czipa et al. 2012), the reduction in this parameter is more obvious in floral samples, in which proline content (which was very high in the unheated sample, 832 mg/kg) was reduced by 38, 42 and 44% at 50, 60 and 80 °C when heated for 20 min. Heating also has an effect on the colour of honey samples that is due to the increase in HMF content. The colour value was increased by 50%, therefore the extra white colour became white. Heating had no significant effect on TPC content, electrical conductivity, pH, moisture content and element contents.

### The effect of sugar syrups

When the honey with added sugar products was tested for physicochemical properties, diastase activities and HMF contents were very low in every sample and GS did not show the presence of diastase (Table 4). Proline contents were also low, except GS where the value was very high. Low TPC content was measured in GS and FGS, but the two IS samples showed a high content. Low electrical conductivity was determined in all four samples. The highest moisture content was determined in the ISE product followed by FGS, ISA and GS. GS and FGS showed higher pH values than ISA and ISE. The highest sucrose content was measured in ISA, but the sucrose content of ISE was very low. Fructose content was low in GS and ISA and the other two sugar product showed similar high values. Glucose content was about 30% in GS, FGS and ISE but ISA showed a much lower value. B, Cd, Cr, Fe, K, Mg, Na and Zn concentrations were under DL in GS and Cr, K, Mg and Zn content were also under DL in FGS. As and S contents were higher in the commercial sugar products, GS and FGS, but the other element content was higher in other two sugar samples.

**Table 4:** Physicochemical parameters of sugar products

Parameters	Adulterants			
	GS	FGS	ISA	ISE
Diastase activity (DN)	< DL	2.20 ± 0.03	1.61 ± 0.04	1.92 ± 0.02
HMF content (mg/kg)	0.82 ± 0.02	1.12 ± 0.05	0.51 ± 0.02	0.56 ± 0.02
Proline content (mg/kg)	236 ± 11	12.0 ± 0.0	33.0 ± 0.58	39.2 ± 1.3
TPC (mgGAE/100 g)	2.19 ± 0.02	8.29 ± 0.11	24.4 ± 1.0	28.0 ± 1.5
EC (µS/cm)	7.49 ± 0.12	11.0 ± 0.2	39.1 ± 0.00	65.2 ± 0.00
Colour (mm)	< DL	< DL	7.4 ± 0.5	6.1 ± 0.2
pH	5.57 ± 0.12	5.26 ± 0.23	4.26 ± 0.01	4.47 ± 0.08
Moisture (%)	14.9 ± 0.1	20.7 ± 0.0	16.2 ± 0.7	25.4 ± 0.3
Sucrose (%)	6.42 ± 0.05	4.45 ± 0.05	69.6 ± 0.6	0.34 ± 0.04
Fructose (%)	7.63 ± 0.03	38.1 ± 1.0	10.6 ± 0.26	34.9 ± 0.3
Glucose (%)	30.6 ± 0.20	30.4 ± 0.3	9.32 ± 0.06	33.7 ± 0.3
As (µg/kg)	42.7 ± 0.2	35.8 ± 0.1	4.48 ± 0.06	4.62 ± 0.12
B (mg/kg)	< DL	< DL	0.37 ± 0.01	0.17 ± 0.02
Cd (µg/kg)	< DL	1.02 ± 0.09	0.66 ± 0.6	0.74 ± 0.03
Cr (µg/kg)	< DL	< DL	< DL	< DL
Fe (µg/kg)	< DL	26.7 ± 0.7	50.4 ± 2.3	86.2 ± 0.9
K (mg/kg)	< DL	< DL	26.5 ± 1.1	24.7 ± 0.9
Mg (mg/kg)	< DL	< DL	0.77 ± 0.03	0.38 ± 0.01
Na (mg/kg)	< DL	1.09 ± 0.03	13.5 ± 0.4	51.5 ± 0.6
P (mg/kg)	0.21 ± 0.01	1.80 ± 0.02	< LOD	1.36 ± 0.02
S (mg/kg)	13.6 ± 0.2	16.8 ± 0.5	6.42 ± 0.25	6.38 ± 0.13
Zn (µg/kg)	< DL	< DL	87.0 ± 1.1	134 ± 1

*GS* glucose syrup, *FGS* fructose-glucose syrup, *ISA* invert sugar 1, *ISE* invert sugar 2

The results of original acacia and adulterated honey samples are shown in Table 5. Due to the low diastase activity of sugar products the adulterated samples showed lower DN values than the original acacia honey, even though the diastase activities did not decrease under the prescribed value. However if the original acacia sample has lower diastase activity these sugar products can diminish the diastase activity to less than 8 DN. The proline content of GS was particularly high and due to this fact the adulterated samples with this sugar product showed high proline concentration. However the value of this parameter was very low in FGS, ISA and ISE; therefore the proline content of adulterated samples with these sugar products showed low concentration that was at the limit value of the EU Directive at levels of 60:40. For TPC there was no change in adulterated acacia honey samples with ISA and ISE because of their relative high TPC. In H:GS = 60:40 and H:FGS = 60:40 the diminution was about 35% and 23%. The EU Directive does not regulate the limits of TPC, so attempts to use this value to prove adulteration are not probable. Lower EC was measured in commercial sugar products than in ISA and ISE, hence there was a reduction in adulterated samples. Electrical conductivity is very low in acacia honey due to the low potassium content (Czipa and Kovács 2014), so this parameter is probably not able to filter out the adulterated acacia honeys. When contaminated with sugar products the change of colour was significant, from extra white to water white. The pH values of sugar products were higher than that of the original acacia sample; therefore the pH value was also higher in the adulterated samples. The moisture content was lower in the acacia samples adulterated with GS and ISA due to the low moisture content of these two sugar products. In the case of the other two sugar products the moisture content was higher, therefore the adulterated acacia samples with FGS and ISE showed increased water content. In the case of the Honey:FGS mixture in the ratio 60:40, the moisture content was higher than the limit of the EU Directive. The sucrose content of the original acacia honey was under the DL (0.2%) and mixing with the sugar products increased the sucrose content in adulterated samples. The highest increase was detected in samples adulterated with ISA, in which case the values exceeded 10%, which is greater than the EU Directive. In the case of samples adulterated with GS and FGS, the sucrose content was higher than in the original samples but it was not higher than the maximum limit of the EU Directive. The sucrose content of honeys adulterated with ISE was under the DL. Significant fructose reduction was measured in samples adulterated with GS and ISA. Glucose content did not change except in honey adulterated with ISA, in which case it reduced by about 10%.

**Table 5:** Physicochemical parameters of original and adulterated acacia honey samples

	Original honey	Honey:GS		Honey:FGS		Honey:ISA		Honey:ISE	
		70:30	60:40	70:30	60:40	70:30	60:40	70:30	60:40
Diastase activity (DN)	25.8 ± 0.3	17.5 ± 0.1	15.4 ± 1.0	19.8 ± 0.1	16.9 ± 0.2	19.1 ± 0.4	15.9 ± 0.4	18.8 ± 0.4	16.1 ± 0.1
HMF content (mg/kg)	0.51 ± 0.02	0.53 ± 0.01	0.55 ± 0.02	0.58 ± 0.01	0.59 ± 0.01	0.50 ± 0.01	0.50 ± 0.02	0.51 ± 0.02	0.49 ± 0.01
Proline content (mg/kg)	284 ± 5	274 ± 4	260 ± 4	209 ± 7	179 ± 3	199 ± 9	181 ± 7	214 ± 6	184 ± 2
TPC (mgGAE/100 g)	20.5 ± 0.2	14.9 ± 0.1	13.3 ± 0.1	16.9 ± 0.1	15.7 ± 0.0	21.9 ± 1.1	22.0 ± 0.1	22.9 ± 1.1	23.3 ± 1.0
EC (µS/cm)	224 ± 3	161 ± 3	137 ± 2	159 ± 3	140 ± 3	159 ± 3	142 ± 3	174 ± 7	153 ± 3
Colour (mm)	12.0 ± 0.2	5.3 ± 0.5	3.99 ± 0.10	3.99 ± 0.03	1.09 ± 0.08	8.42 ± 0.15	7.11 ± 0.08	7.93 ± 0.11	7.02 ± 0.10
pH	3.91 ± 0.01	4.48 ± 0.01	4.63 ± 0.01	4.03 ± 0.01	4.52 ± 0.02	4.04 ± 0.17	4.10 ± 0.06	4.10 ± 0.07	4.16 ± 0.11
Moisture (%)	16.5 ± 0.1	16.2 ± 0.1	15.9 ± 0.1	17.9 ± 0.1	18.1 ± 0.3	16.4 ± 0.4	16.4 ± 0.4	19.5 ± 0.1	20.2 ± 0.2
Sucrose (%)	< DL	2.12 ± 0.41	2.73 ± 0.12	1.41 ± 0.11	1.82 ± 0.21	18.7 ± 0.4	25.8 ± 1.1	< DL	< DL
Fructose (%)	47.3 ± 1.1	36.2 ± 1.5	30.3 ± 1.1	44.2 ± 1.3	43.8 ± 1.8	35.4 ± 1.2	31.8 ± 0.2	43.4 ± 1.4	42.9 ± 1.1
Glucose (%)	32.7 ± 1.3	32.5 ± 1.0	32.4 ± 1.2	32.4 ± 1.1	32.4 ± 1.4	24.1 ± 0.2	22.6 ± 1.6	33.5 ± 1.1	33.6 ± 0.4
As (µg/kg)	38.2 ± 1.2	38.2 ± 1.3	40.3 ± 1.3	37.4 ± 1.5	37.3 ± 2.1	27.3 ± 1.3	25.1 ± 0.8	29.2 ± 0.7	22.3 ± 0.9
B (mg/kg)	5.39 ± 0.16	3.82 ± 0.12	3.05 ± 0.12	3.82 ± 0.25	3.13 ± 0.04	3.91 ± 0.51	3.42 ± 0.04	3.74 ± 0.12	3.25 ± 0.05
Cd (µg/kg)	1.03 ± 0.12	0.61 ± 0.02	0.62 ± 0.03	1.03 ± 0.02	1.02 ± 0.09	1.01 ± 0.03	0.83 ± 0.05	0.98 ± 0.02	0.92 ± 0.05
Cr (µg/kg)	< DL	< DL	< DL	< DL	< DL	< DL	< DL	< DL	< DL
Fe (µg/kg)	691 ± 15	482 ± 12	429 ± 9	501 ± 8	420 ± 12	502 ± 4	431 ± 8	508 ± 5	442 ± 4
K (mg/kg)	267 ± 3	182 ± 3	158 ± 5	193 ± 3	158 ± 4	198 ± 3	164 ± 5	201 ± 7	172 ± 9
Mg (mg/kg)	6.22 ± 0.15	4.48 ± 0.05	3.79 ± 0.1	4.38 ± 0.34	3.73 ± 0.2	4.69 ± 0.14	4.13 ± 0.11	4.39 ± 0.08	3.98 ± 0.14
Na (mg/kg)	4.74 ± 0.11	3.35 ± 0.02	2.86 ± 0.07	3.65 ± 0.56	3.26 ± 0.06	7.51 ± 0.21	8.41 ± 0.05	20.1 ± 0.11	22.3 ± 0.01
P (mg/kg)	37.6 ± 1.4	26.8 ± 0.1	22.7 ± 0.07	26.1 ± 0.6	23.6 ± 0.37	26.1 ± 0.2	22.4 ± 0.4	26.2 ± 1.1	22.9 ± 0.7
S (mg/kg)	20.8 ± 1.5	18.2 ± 0.1	18.0 ± 0.1	18.9 ± 1.2	19.0 ± 0.1	16.3 ± 0.1	15.2 ± 0.3	16.5 ± 0.7	15.2 ± 0.1
Zn (µg/kg)	864 ± 14	612 ± 12	503 ± 20	598 ± 11	509 ± 13	611 ± 7	547 ± 3	664 ± 5	565 ± 8

Since B, Cd, Cr, Fe, K, Mg, Na and Zn were not detected in GS, the concentration of these elements in adulterated acacia honey samples with a 60:40 ratio was lower by about 40% than in the original sample, similarly with the B, K, Mg and Zn content of samples adulterated with FGS. Arsenic content was not changed in acacia honey samples adulterated with either GS or FGS; however the concentration of this toxic element decreased in samples adulterated with ISA and ISE. Cadmium was detected in both original acacia honey samples and sugar products—except in GS—and these concentrations were similar, thus adulteration did not bring about an important change. In the case of other elements some reduction was observed in adulterated acacia samples.

### The effect of collection year and location

After LDA and LSD tests, it was concluded that there was no statistically verifiable difference due to collecting years. However, examining the collecting area (Eastern Hungary and Western Hungary), there were statistically verified differences in Na, Fe and As concentrations, according to an Independent-Samples T Test. Acacia honeys from Eastern Hungary showed higher Na (3.65 ± 1.19 mg/kg) and Fe (0.308 ± 0.173 mg/kg) concentrations and lower As (12.8 ± 6.73 µg/kg) concentration than acacia honeys from Western Hungary (2.64 ± 0.56 mg/kg for Na, 0.166 ± 0.170 mg/kg for Fe and 18.0 ± 6.45 µg/kg for As, respectively).

### The effect of storage

In Hungary the maximum durability of honeys is 2 years. Because of this, the changes in different parameters of five acacia honey samples were determined 2 years after collection. Table 6 contains parameters which showed changes under storage. No change was measured in electrical conductivity, pH, moisture and element contents of samples. Corresponding with the existing literature (White 2000), the diastase activities were lower and HMF contents higher after storage. The reduction was not important in the case of diastase activity, however increases of HMF contents were more significant. Lower proline contents and higher TPCs were measured after storage. Small increases were measured in sucrose content. Colour of samples 1, 3, 4 and 5 was water white (< 9) immediately after collecting. Samples 1, 4 and 5 showed a darker colour (13 mm, 15 mm and 13 mm, respectively) after 2 years. The colour value of Sample 3 was less than 9 mm after storage. Sample 2 showed a darker colour with 16 mm (after collecting) and with 30 mm (after 2 years).

**Table 6:** Physicochemical parameters of acacia honeys after 2 years storage

	Diastase activity (DN)	HMF content (mg/kg)	Proline content (mg/kg)	TPC (mgGAE/100 g)	Sucrose (%)	Color (mm)
Sample 1						
2015	17.1 ± 0.3	1.05 ± 0.01	242 ± 5	20.3 ± 0.2	0.52 ± 0.02	Water white
2017	14.3 ± 0.2	12.1 ± 0.1	230 ± 4	25.3 ± 0.1	0.78 ± 0.01	Extra white
Sample 2						
2015	16.8 ± 0.3	4.27 ± 0.51	290 ± 2	19.0 ± 0.2	1.13 ± 0.03	Extra white
2017	14.2 ± 0.3	20.2 ± 0.8	261 ± 3	24.4 ± 0.1	1.56 ± 0.01	White
Sample 3						
2015	15.7 ± 0.1	0.451 ± 0.012	280 ± 4	19.1 ± 0.2	0.69 ± 0.03	Water white
2017	14.0 ± 0.2	9.41 ± 0.12	263 ± 2	23.5 ± 0.2	0.98 ± 0.02	Water white
Sample 4						
2015	20.1 ± 0.0	1.80 ± 0.05	244 ± 3	17.3 ± 0.1	1.69 ± 0.12	Water white
2017	16.7 ± 0.1	13.4 ± 0.2	219 ± 4	21.5 ± 0.3	2.03 ± 0.19	Extra white
Sample 5						
2015	19.3 ± 0.1	1.87 ± 0.10	222 ± 3	16.2 ± 0.2	0.88 ± 0.03	Water white
2017	16.1 ± 0.1	12.9 ± 0.7	196 ± 4	20.2 ± 0.1	1.23 ± 0.11	Extra white

## Conclusion

Centrifugation and filtration reduced the proline, TPC, electrical conductivity and examined element content of honey samples, which can be ascribed to removal of fragments of the wax capping and comb pieces. Heating had no effect on the mineral content of acacia honeys, but reduced diastase activity and increased HMF at high temperatures. It also reduced total phenolic content and proline content, and increased the colour to a small extent. Honey samples adulterated with sugar additives had reduced diastase activity, proline content, TPC (except for ISA and ISE), electrical conductivity, colour, fructose content, B, Fe, K, Mg, P, S and Zn contents, compared with the original acacia honey samples. These adulterated samples had increased pH, moisture (except for FGS and ISE) and sucrose (except for ISE) than the original samples. The adulterant used did not change the values of HMF, glucose (except for SA), As (except for ISA and ISE) or Cd content. Year of collection did not have any effect on the examined physicochemical parameters of acacia honeys, however, the collecting area affected the Na, Fe and As concentrations. Two years storage did not have major effects on the physicochemical properties of acacia honeys, but it reduced diastase activity and proline content and increased HMF content, TPC and colour values.
